# Prognostic Factors Associated With Mortality in Adult Hemophagocytic Lymphohistiocytosis: A Systematic Review and Meta-Analysis

**DOI:** 10.7759/cureus.113630

**Published:** 2026-07-29

**Authors:** Shaza Bakri Osman Elsayed, Hoyam Atitalla, Salma Elrayah Yousif Ahmed, Mohamed Kamal Gomaa Abdelfattah, Mahmoud Abdelghany Eldyasty Ahmed Eid, Lobaba Mubarak Saidahmed Ahmed, Eltayeb Osman Elfaki Omer, Mohamed Sobhy Mahmoud Rezk

**Affiliations:** 1 Rheumatology, Croydon Health Services NHS Trust, London, GBR; 2 Rheumatology, Basingstoke and North Hampshire Hospital, Hampshire Hospitals NHS Foundation Trust, Basingstoke, GBR; 3 Internal Medicine, MedicFirst, Melbourne, AUS; 4 Rheumatology, King Salman Specialist Hospital, Hail, SAU; 5 Dermatology, Al-Azhar University, Cairo, EGY; 6 Internal Medicine, Shendi University, Riyadh, SAU; 7 Endocrinology, Dr. Sulaiman Al Habib Hospital, Riyadh, SAU; 8 Rheumatology, Faculty of Medicine, Al-Azhar University, Cairo, EGY

**Keywords:** ferritin, hemophagocytic lymphohistiocytosis, malignancy, meta-analysis, mortality, prognosis, systematic review

## Abstract

Adult hemophagocytic lymphohistiocytosis (HLH) carries a high case-fatality rate, yet reported prognostic factors are discordant and have never been pooled. The aim of this study was to perform the first systematic review and meta-analysis of prognostic factors for mortality in adult HLH. PubMed/MEDLINE, Embase, Web of Science, and the Cochrane Library were searched (from database inception to February 15, 2026) for observational cohort studies of adults (≥18 years) with HLH diagnosed by the HLH-2004 criteria and/or the HScore, reporting mortality against at least one candidate prognostic factor. Risk of bias was appraised using the Quality In Prognosis Studies (QUIPS) tool, and certainty using GRADE. Random-effects meta-analyses (DerSimonian-Laird with Hartung-Knapp correction) were pre-specified for factors reported in at least three cohorts. Reporting followed Preferred Reporting Items for Systematic Reviews and Meta-Analyses (PRISMA) 2020.

A total of 18 cohorts (3,570 adults) were included. Pooled mortality was 49.5% (95% CI: 41.1-57.8), rising from 34.4% (29.0-40.0) within 30 days to 57.3% (47.9-66.4) at longest follow-up (p<0.001). Malignancy-associated HLH carried higher mortality than other triggers (47.1% vs. 32.2%; 12 cohorts, n=2,373; pooled odds ratio 2.53, 95% CI: 1.55-4.12; I²=66.7%). Older age (2.23, 95% CI: 1.57-3.15; I²=0%), hyperferritinemia (3.07, 95% CI: 1.07-8.82), and elevated lactate dehydrogenase (2.02, 95% CI: 1.05-3.88) independently predicted death; thrombocytopenia and prolonged activated partial thromboplastin time were directionally harmful but imprecise. Egger’s test indicated small-study effects (p=0.014). Certainty was low to moderate. Approximately half of adults with HLH die following diagnosis, with nearly one-third of deaths occurring within 30 days. A malignant trigger approximately doubles the odds of death; age, ferritin, and lactate dehydrogenase add independent information. Prospective, multinational registry data with pre-specified thresholds are needed.

## Introduction and background

Hemophagocytic lymphohistiocytosis (HLH) is a syndrome of uncontrolled immune activation in which defective cytotoxic killing by natural killer cells and CD8-positive T lymphocytes fails to terminate an antigenic stimulus, producing sustained hypercytokinemia, tissue infiltration by activated macrophages, and progressive multiorgan failure [1]. The diagnostic architecture in universal use, the eight-item HLH-2004 criteria, was derived and validated exclusively in children with familial disease [1], yet the overwhelming majority of adults who fulfill those criteria have an acquired, secondary syndrome precipitated by malignancy, infection, or an autoimmune disorder [2]. This mismatch between the pediatric provenance of the diagnostic framework and the adult population to which it is applied has been repeatedly identified as a central obstacle in the field, and it has prompted the development of alternative, adult-oriented instruments such as the HScore [3,4].

The HLH-2004 criteria combine clinical, laboratory, and, where familial disease is suspected, genetic variables (fever, splenomegaly, bi- or tri-lineage cytopenia, hypertriglyceridemia and/or hypofibrinogenemia, tissue hemophagocytosis, low or absent natural killer cell activity, hyperferritinemia, and elevated soluble CD25) and a diagnosis requires five of these eight items or a confirmed causative mutation [1]. The HScore, by contrast, assigns weighted points to nine readily available clinical and laboratory variables - known underlying immunosuppression, temperature, organomegaly, number of cytopenias, and levels of ferritin, triglycerides, fibrinogen, aspartate aminotransferase, and the presence of hemophagocytosis on marrow examination - to estimate the probability of reactive hemophagocytic syndrome without requiring specialized assays [5]. Adult HLH remains, nevertheless, a disease diagnosed late, treated empirically, and frequently fatal.

Clinically, adult HLH typically presents as a febrile, acutely unwell patient with hepatosplenomegaly, progressive bi- or tri-lineage cytopenia, deranged liver function and coagulation, and a markedly elevated serum ferritin, often progressing to multiorgan dysfunction and admission to intensive care. Because these features overlap with sepsis, disseminated malignancy, and severe autoimmune flares, the diagnosis is frequently delayed. Reliable prognostic information matters precisely at this point of uncertainty as follows: an early, evidence-based estimate of the risk of death helps the clinician decide how aggressively to pursue an underlying trigger, whether to commit a cytopenic and hemodynamically fragile patient to cytotoxic immunochemotherapy, and how to counsel the family. The prognostic determinants in adults differ from those in children because the underlying biology differs - pediatric disease is dominated by inherited defects of lymphocyte cytotoxicity, whereas adult disease is overwhelmingly an acquired reaction to malignancy, infection, or autoimmunity - so prognostic models derived in children cannot simply be transferred to adults.

The therapeutic corollary of this diagnostic uncertainty is that adults are treated with regimens designed for children. The HLH-94 protocol of etoposide and dexamethasone, which transformed the outlook for pediatric familial disease and achieved a five-year survival of approximately 54% in that population, is routinely extrapolated to adults, in whom the underlying biology, the spectrum of triggers, and the tolerance of cytotoxic therapy are fundamentally different [3,6]. Reported adult case-fatality rates span an extraordinarily wide range, from roughly one-quarter to more than three-quarters of patients, depending on the referral setting, the dominant trigger, and the length of follow-up [2,7]. In critically ill adults, in whom the syndrome is most often encountered by intensivists, the diagnostic performance of both the HLH-2004 criteria and the HScore is modest, and mortality approaches 60% [8]. The practical consequence is that clinicians confronting an adult with suspected HLH must decide, often within hours and on incomplete information, whether to commit a cytopenic and hemodynamically fragile patient to etoposide-based immunochemotherapy, to treat the underlying trigger alone, or to escalate to salvage strategies [9]. That decision would be substantially better informed by a reliable estimate of baseline mortality risk.

A considerable body of retrospective work has attempted to supply exactly that estimate. Individual cohorts have nominated a malignant trigger, older age, thrombocytopenia, hypoalbuminemia, hyperferritinemia, coagulopathy, and elevated lactate dehydrogenase as independent predictors of death, and several have assembled these variables into prognostic indices or nomograms. Yet the primary literature is fragmented as follows: cohorts are small; almost all are single-center and retrospective; the reported cut-points are optimized within each dataset and consequently differ by more than an order of magnitude between studies; and the adjustment sets used in multivariable modeling are neither pre-specified nor consistent. Narrative and scoping syntheses have cataloged which factors are reported most often, and large reactive-HLH series have described prognosis in aggregate, but no study has quantitatively pooled effect estimates across cohorts, and consequently the field lacks a synthesized, precision-weighted answer to the most basic prognostic question in adult HLH as follows: which variables measurable at diagnosis actually predict death, and by how much? [4,7].

We therefore conducted a systematic review and meta-analysis of prognostic factors associated with mortality in adult HLH, with the objectives of (i) estimating pooled early and overall mortality, (ii) quantifying the association between each candidate prognostic factor and death, (iii) exploring the sources of between-study heterogeneity, and (iv) formally rating the certainty of the resulting evidence.

## Review

Materials and methods

Study Design

This systematic review and meta-analysis was designed, conducted, and reported in accordance with the Preferred Reporting Items for Systematic Reviews and Meta-Analyses (PRISMA) 2020 statement [10]. The review was not prospectively registered with PROSPERO, a limitation acknowledged in the limitations subhead. Because only aggregate data from previously published reports were analyzed, institutional review board approval and informed consent were not required.

Eligibility Criteria

Eligibility was framed using the PICOS structure and is set out in full in Table 1. In brief, we included original observational cohort studies of adults aged 18 years or older with HLH diagnosed by the HLH-2004 criteria and/or the HScore that reported all-cause mortality in relation to at least one candidate prognostic factor and from which either a multivariable-adjusted effect estimate with its 95% confidence interval or a two-by-two table of deaths by exposure category could be extracted. Reviews, conference abstracts, preprints, case series of fewer than 20 patients, pediatric and non-separable mixed-age cohorts, single-trigger sub-populations, administrative-database studies without verification against diagnostic criteria, and reports published in journals delisted for research-integrity concerns were excluded. No language or date restriction was applied.

**Table 1:** Eligibility criteria according to the PICOS framework. PICOS: Population, Intervention, Comparison, Outcomes, and Study design; HLH: hemophagocytic lymphohistiocytosis; LDH: lactate dehydrogenase

PICOS domain	Inclusion criteria	Exclusion criteria
Population (P)	Adults ≥18 years with HLH diagnosed by HLH-2004 criteria (≥5 of 8 items or a confirmed causative mutation) and/or an HScore indicating reactive hemophagocytic syndrome; any underlying trigger (malignancy, infection, autoimmune disease, idiopathic).	Exclusively pediatric cohorts; mixed-age cohorts in which adult data cannot be separated; cohorts restricted to a single trigger (e.g. malignancy-associated HLH only or HLH complicating one named infection); HLH ascertained solely from administrative codes without verification against diagnostic criteria.
Prognostic factor (I)	Any demographic, clinical, or laboratory variable measured at or around the time of HLH diagnosis and evaluated as a candidate predictor of death (e.g., age, sex, underlying trigger, platelet count, ferritin, albumin, LDH, coagulation indices, cytokines).	Variables measured only after completion of therapy, where the timing precludes an inference about baseline prognosis; treatment received, when analyzed as the sole exposure of interest.
Comparator (C)	Patients without the prognostic factor, or in the reference category of the exposure (e.g. non-malignancy-associated HLH; ferritin below the study threshold).	No comparator group; single-arm descriptive reports without a contrast.
Outcome (O)	All-cause mortality, reported as early mortality (≤30 days or in-hospital) and/or mortality at longest follow-up, with either a multivariable-adjusted ratio measure and 95% CI, or a two-by-two table of deaths by exposure category.	No mortality outcome reported; mortality reported without any link to a prognostic factor; outcome reported only as a composite that cannot be decomposed.
Study design (S)	Original observational cohort studies (prospective, retrospective, or bidirectional), single-center or multicenter, with ≥20 adult participants; peer-reviewed and fully published.	Reviews, systematic reviews, scoping reviews, meta-analyses, editorials, commentaries; conference abstracts; preprints; case reports and case series of <20 patients; studies published in journals delisted for research-integrity concerns; articles for which the full text is unobtainable.

Information Sources and Search Strategy

PubMed/MEDLINE, Embase, Web of Science Core Collection, and the Cochrane Library were searched from database inception through the last search date of February 15, 2026, using both controlled vocabulary and free-text terms for the population and outcome. The core PubMed strategy was as follows: ("hemophagocytic lymphohistiocytosis"[MeSH] OR "haemophagocytic lymphohistiocytosis"[tiab] OR "hemophagocytic syndrome"[tiab] OR "haemophagocytic syndrome"[tiab] OR HLH[tiab] OR "macrophage activation syndrome"[tiab]) AND (prognos*[tiab] OR predict*[tiab] OR "risk factor*"[tiab] OR mortality[tiab] OR survival[tiab] OR death[tiab] OR outcome*[tiab]) AND (adult*[tiab] OR "adults"[MeSH]). This strategy was translated for each remaining database using the appropriate syntax. Reference lists of all included articles and of relevant reviews were hand-searched, and forward citation tracking was performed.

Study Selection and Data Extraction

Records were de-duplicated in EndNote and screened independently and in duplicate, first by title and abstract and then by full text; disagreements were resolved by discussion and, where necessary, by adjudication from a third reviewer. Data were extracted into a standardized, piloted form, including first author, year, country, recruitment period, design and setting, sample size, diagnostic criteria, age, sex, distribution of underlying triggers, treatment received, the definition and timing of the mortality outcome, deaths overall and by trigger category, and - for every candidate prognostic factor - the reported threshold, the univariable and multivariable estimates with 95% confidence intervals, and the covariates entered into the model. Where a study reported both a derivation and a validation cohort, data from both were combined for descriptive purposes, but only the derivation cohort was used for effect-estimate pooling, to avoid double counting.

Outcomes

The primary outcome was all-cause mortality. Because the primary studies used heterogeneous outcome windows, we pre-specified two strata as follows: early mortality (death within 30 days of diagnosis, or in-hospital death) and overall mortality (death at the longest follow-up interval reported by each study).

Risk of Bias and Certainty of Evidence

Risk of bias was appraised with the Quality In Prognosis Studies (QUIPS) tool, the instrument recommended by the Cochrane Prognosis Methods Group for reviews in which the unit of analysis is a prognostic factor-outcome association [11]. Each study was rated low, moderate, or high risk in the following six domains: study participation, study attrition, prognostic factor measurement, outcome measurement, study confounding, and statistical analysis and reporting. The Newcastle-Ottawa scale was considered and rejected because it contains no domain addressing prognostic factor measurement and none addressing selective analysis reporting - the two biases that most plausibly threaten this literature, in which cut-points are almost universally optimized within the analysis dataset. Assessments were made independently by two reviewers and reconciled by consensus. The certainty of the body of evidence for each pooled factor was rated using GRADE as adapted for prognostic factor reviews, in which observational cohort evidence begins at high certainty and is downgraded for risk of bias, inconsistency, indirectness, imprecision, and publication bias [12].

Statistical Analysis

A prognostic factor was pooled only if a compatible estimate could be extracted from at least three independent cohorts, a rule fixed in advance to prevent post-hoc selection of factors on the basis of their apparent significance. For the underlying trigger, unadjusted odds ratios were computed from each study’s two-by-two table with a 0.5 continuity correction where a zero cell arose; for all other factors, the multivariable-adjusted hazard or odds ratios reported by the primary authors were combined on the natural-logarithm scale as ratio measures of association, with reciprocals taken where an estimate was reported in the protective direction. Hazard ratios and odds ratios were pooled together as generic ratio measures of association because the primary studies reported a mixture of the two and individual-participant time-to-event data were unavailable; the resulting summary estimates should therefore be interpreted as indicating the strength and direction of the association with death rather than as exact hazard or risk ratios. Because mortality in this population is common rather than rare, the odds ratios overstate the corresponding risk ratios, and this approximation biases the odds-ratio-based contrasts away from the null; this is quantified qualitatively in the limitations. All syntheses used a random-effects DerSimonian-Laird model with the Hartung-Knapp variance correction, which is markedly more reliable than the normal approximation when few studies contribute; classical DerSimonian-Laird intervals are reported as a sensitivity analysis [13]. Mortality proportions were pooled after Freeman-Tukey double-arcsine transformation. Heterogeneity was quantified by Cochran’s Q, τ² and I², and 95% prediction intervals were derived [14]. Pre-specified subgroup analyses examined geographical region, mortality window, and cohort size; robustness was tested using a leave-one-out analysis; and small-study effects were assessed using a funnel plot and Egger’s regression test in the single analysis containing at least 10 studies [15]. Tests were two-sided (α=0.05). Analyses were performed in Python 3.12 (NumPy, SciPy).

Results

Study Selection

**Figure 1:**
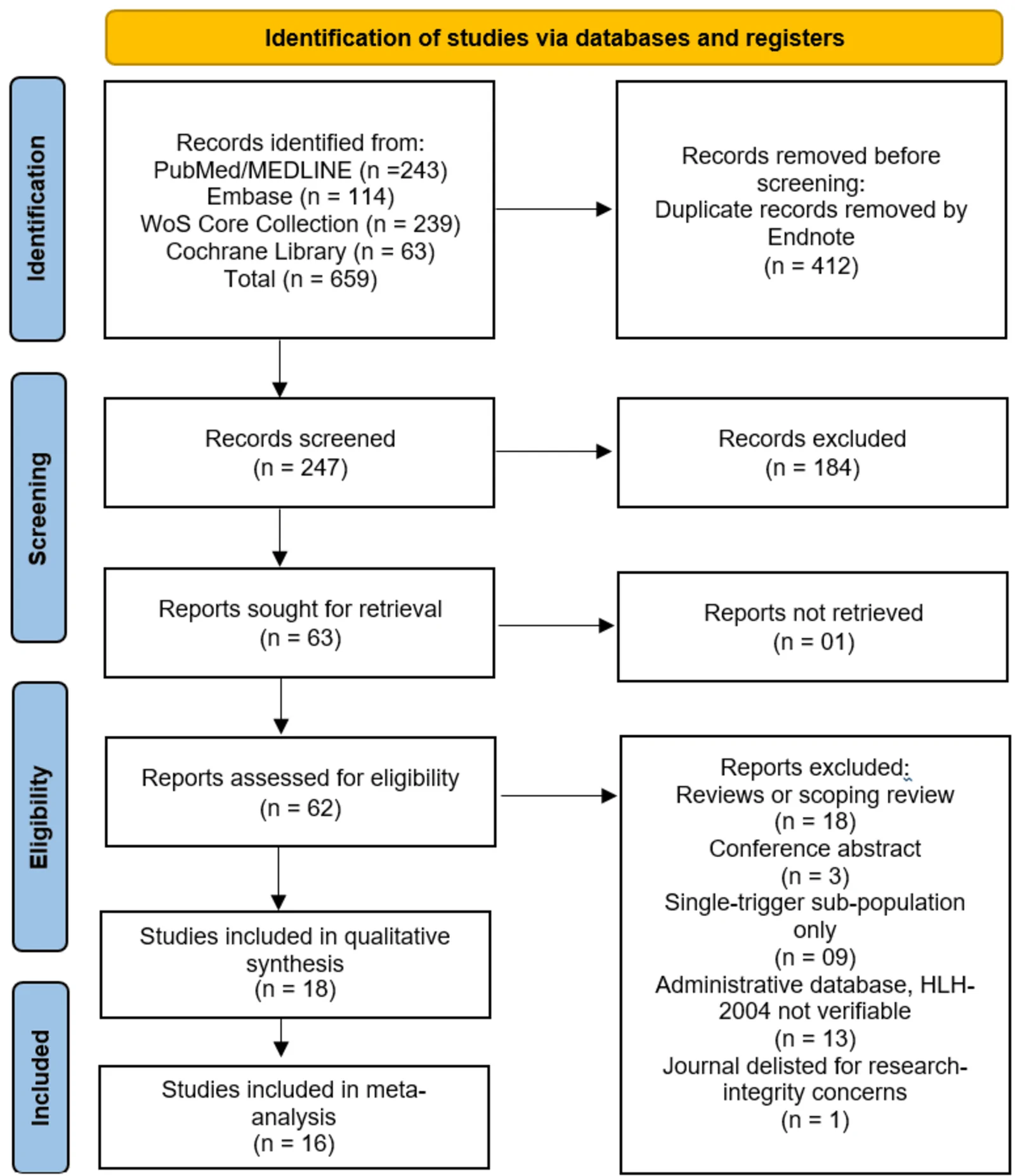
PRISMA 2020 flow diagram of study identification, screening, and inclusion. PRISMA: Preferred Reporting Items for Systematic Reviews and Meta-Analyses

The study-selection process is summarized in Figure 1. The electronic search retrieved 659 records (PubMed/MEDLINE: 243, Embase: 114, Web of Science Core Collection: 239, and Cochrane Library: 63), from which 412 duplicates were removed in EndNote, leaving 247 records for title and abstract screening. Of these, 184 were excluded as irrelevant, and 63 reports were sought for retrieval; one could not be obtained, leaving 62 full-text reports assessed for eligibility. A total of 44 reports were excluded as follows: 18 reviews or scoping reviews, 13 administrative-database cohorts in which HLH-2004 ascertainment could not be verified, nine studies confined to a single etiological sub-population, three conference abstracts, and one report published in a journal subsequently delisted for research-integrity concerns. A total of 18 studies, comprising 3,570 adults with HLH, met all eligibility criteria and were included in the qualitative synthesis; 16 of these contributed data to at least one meta-analysis [16-33].

Characteristics of Included Studies

The characteristics of the 18 included cohorts are presented in Table 2. All studies were observational - 15 were single-center retrospective series, two were multicenter or two-center retrospective analyses [25,29], and one was a bidirectional observational cohort [31]. A total of 15 studies were conducted in China [18,20-27,29-33], two in the United States [16,17], and one each in Italy and Belgium [19,28]. Cohort size ranged from 35 to 711 patients, with a median of 163 [19,32]. Median age at diagnosis clustered between 41 and 58 years, and men were the majority in most series. All studies applied the HLH-2004 criteria; three supplemented or corroborated this with the HScore [19,20,28]. Notably, soluble CD25 and natural killer cell activity were unavailable in the majority of the Chinese centers, such that diagnosis in those cohorts rested on five of the remaining six criteria, an ascertainment nuance with obvious implications for comparability.

**Table 2:** Characteristics of the 18 included studies. FU: follow-up; OS: overall survival

Studies	Country	Design	n	Age	Malignancy trigger, n (%)	Deaths, n (%)	Outcome window
Parikh et al. (2014) [16]	USA	Single-center retrospective	62	49	32 (52)	41 (66)	Overall (median FU 42 months)
Otrock and Eby (2015) [17]	USA	Single-center retrospective	73	51	20 (27)	38 (52)	Overall (1-year OS 48%)
Li et al. (2015) [18]	China	Single-center retrospective	85	44	23 (27)	39/60 (65)	Overall
Cattaneo et al. (2017) [19]	Italy	Single-center retrospective	35	54	24 (69)	26/34 (76)	Overall (median FU 649 days)
Zhang et al. (2018) [20]	China	Single-center retrospective	174	51	92 (53)	102 (59)	Overall (5-year OS 41.8%)
Zhao et al. (2019) [21]	China	Single-center retrospective	171	49	88 (52)	61 (36)	Early (30 days)
Zhou et al. (2020) [22]	China	Two-cohort retrospective	229	49	53 (23)	Not extractable	Overall (1-year OS 36.6%)
Zhou et al. (2020) [23]	China	Single-center retrospective	136	50	38 (28)	71 (52)	6 months
Lu et al. (2021) [24]	China	Two-cohort retrospective	155	49	16 (10)	69 (45)	1 year
Shen et al. (2022) [25]	China	Multicenter retrospective (8 centers)	270	56	97 (36)	Not extractable	Overall (5-year OS)
Wang et al. (2022) [26]	China	Single-center retrospective	204	44	125 (61)	70 (34)	90 days
Zhou et al. (2022) [27]	China	Single-center retrospective	431	52	80 (19)	128 (30)	Early (30 days)
Yildiz et al. (2022) [28]	Belgium	Single-center retrospective	52	48	22 (42)	24 (46)	Overall
Zhang et al. (2023) [29]	China	Two-center retrospective	324	50	145 (45)	134 (41)	Early (30 days)
Qiu et al. (2023) [30]	China	Two-cohort retrospective	142	58	19 (26)	49/72 (68)	Overall (median OS 102 days)
Pei et al. (2024) [31]	China	Bidirectional cohort	220	41	73 (33)	71 (32)	Early (in-hospital)
Xie et al. (2025) [32]	China	Single-center retrospective	711	56	326 (46)	241 (34)	Early (30 days)
Ling et al. (2026) [33]	China	Single-center retrospective	96	45	47 (49)	69 (72)	Overall

The distribution of underlying triggers differed systematically by geography. Malignancy predominated in the East Asian and Italian hematology-based series, accounting for 61.3% of cases in one West China cohort, 52.9% in a Zhejiang hematology cohort, and 69% in the Italian hematology unit [19,20,26]; infection predominated in the two North American series and in several Chinese cohorts recruited through laboratory medicine or infectious disease departments [17,24,27]. Two cohorts derived from rheumatology-facing services reported macrophage activation syndrome as the leading trigger [31]. The multivariable predictors nominated by each study are collated in Table 3 and illustrate the central problem this review set out to address as follows: 22 distinct variables were reported as independent predictors of death across the 18 cohorts, and the thresholds applied to any single continuous variable varied enormously - the ferritin cut-point, for example, ranged from 2,000 µg/L to 50,000 µg/L, a 25-fold span, and the age cut-point from 25 years to 60 years [17,26,29].

**Table 3:** Independent predictors of mortality identified on multivariable analysis in each included study. *Post-treatment (discharge) ferritin, measured after HLH-directed therapy had begun rather than at diagnosis. These two estimates did not satisfy the eligibility requirement for a baseline prognostic marker and were therefore excluded from the ferritin meta-analysis (see “adjusted prognostic factors” and the discussion); they are shown here only to describe the predictors reported by each study [22,23]. APTT: activated partial thromboplastin time; ALT: alanine aminotransferase; PT: prothrombin time; AST: aspartate aminotransferase; EBV: Epstein-Barr virus; NHL: Non-Hodgkin's lymphoma; OS: overall survival; HLH: hemophagocytic lymphohistiocytosis; LDH: lactate dehydrogenase

Studies	Model and outcome	Independent predictors (effect estimate; 95% CI where reported)
Parikh et al. (2014) [16]	Cox, overall survival	Low serum albumin (p<0.001); malignant trigger (p=0.01)
Otrock and Eby (2015) [17]	Logistic, OS and 30-day mortality	Malignancy (HR: 12.22, 2.53-59.02); ferritin >50,000 µg/L for 30-day death (HR: 3.26, 1.01-10.26)
Li et al. (2015) [18]	Cox, overall survival	Platelets <40×10⁹/L (only independent predictor)
Cattaneo et al. (2017) [19]	Cox, HLH-free survival	Edema (HR: 6.9, 2.0-23.5); bilirubin >2 mg/dL (HR: 6.2, 2.1-18.2); B-cell NHL protective (HR: 0.2)
Zhang et al. (2018) [20]	Cox, overall survival	T-cell NHL (HR: 1.58, 1.03-2.43); lymphocytopenia (HR: 1.69, 1.06-2.69); hypofibrinogenemia (HR: 1.93, 1.11-3.34)
Zhao et al. (2019) [21]	Cox, 30-day mortality	Age ≥54 years (2.34); platelets ≤39.5×10⁹/L (2.36); APTT ≥54 s (1.70); triglycerides ≥3.23 mmol/L (2.89); LDH ≥1,300 U/L (1.97); malignancy (3.21)
Zhou et al. (2020) [22]	Cox, 1-year OS	Post-treatment ferritin* >1,050 µg/L (HR: 3.18, 1.47-6.87); ANC; platelets; ALT; calcium
Zhou et al. (2020) [23]	Logistic, 6-month mortality	Post-treatment ferritin* (0.10); platelets (4.80); ALT (0.42)
Lu et al. (2021) [24]	Logistic, 1-year mortality	Male sex (5.53); altered mental status (11.88); ferritin ≥31,381 µg/L (8.27); IL-6 ≥18.59 pg/mL (19.45)
Shen et al. (2022) [25]	Cox, overall survival	Age (1.54); creatinine (1.40); albumin (0.65); platelets (0.78); lymphocyte ratio (1.33); ALT (1.72)
Wang et al. (2022) [26]	Cox, 90-day OS	Age ≥25 years (2.44); coagulopathy (2.52); hemoglobin (0.32); AST (0.41); LDH ≥687 U/L (2.60); creatinine (4.13); PT ≥17.1 s (2.35); ferritin >2,000 µg/L (3.35)
Zhou et al. (2022) [27]	Logistic, 30-day mortality	Age >47 years (2.99); ferritin >3,678 µg/L (4.26); lymphocytes (0.39); INR (2.51); thrombin time (2.08); globulin (0.51); uric acid (2.64); chloride (0.48)
Yildiz et al. (2022) [28]	Logistic, overall mortality	Age per year (OR: 1.05, 1.02-1.09) - the only variable retaining significance
Zhang et al. (2023) [29]	Logistic, early death	Age >60 years (1.90); platelets ≤20×10⁹/L (1.45); APTT >36 s (1.65); LDH >1,000 U/L (1.92)
Qiu et al. (2023) [30]	Cox, overall survival	IL-10 (HR: 1.002, 1.000-1.003); prothrombin time (HR: 1.26, 1.09-1.47)
Pei et al. (2024) [31]	Logistic, in-hospital death	Age ≥38 years (2.31); cytopenia ≥2 lineages (5.83); platelets ≤50×10⁹/L (3.10); AST ≥135 U/L (2.63); PT ≥14.9 s (3.32); APTT ≥38.5 s (2.77); EBV infection (2.68); fungal infection (2.60)
Xie et al. (2025) [32]	Logistic, 30-day mortality	Age (1.03); ferritin (1.000); APTT (1.02); ALT (1.001); urea (1.11); phosphate (0.44); chloride (0.96)
Ling et al. (2026) [33]	Cox, overall survival	Hemoglobin <70 g/L (2.61); CD3⁺ <50% (4.22); CD3⁻ CD16/CD56⁺ <2% (3.40); ferritin ≥10,000 ng/mL (2.60); splenomegaly (1.75); lymphoma/EBV trigger (0.42)

Pooled Mortality

**Figure 2:**
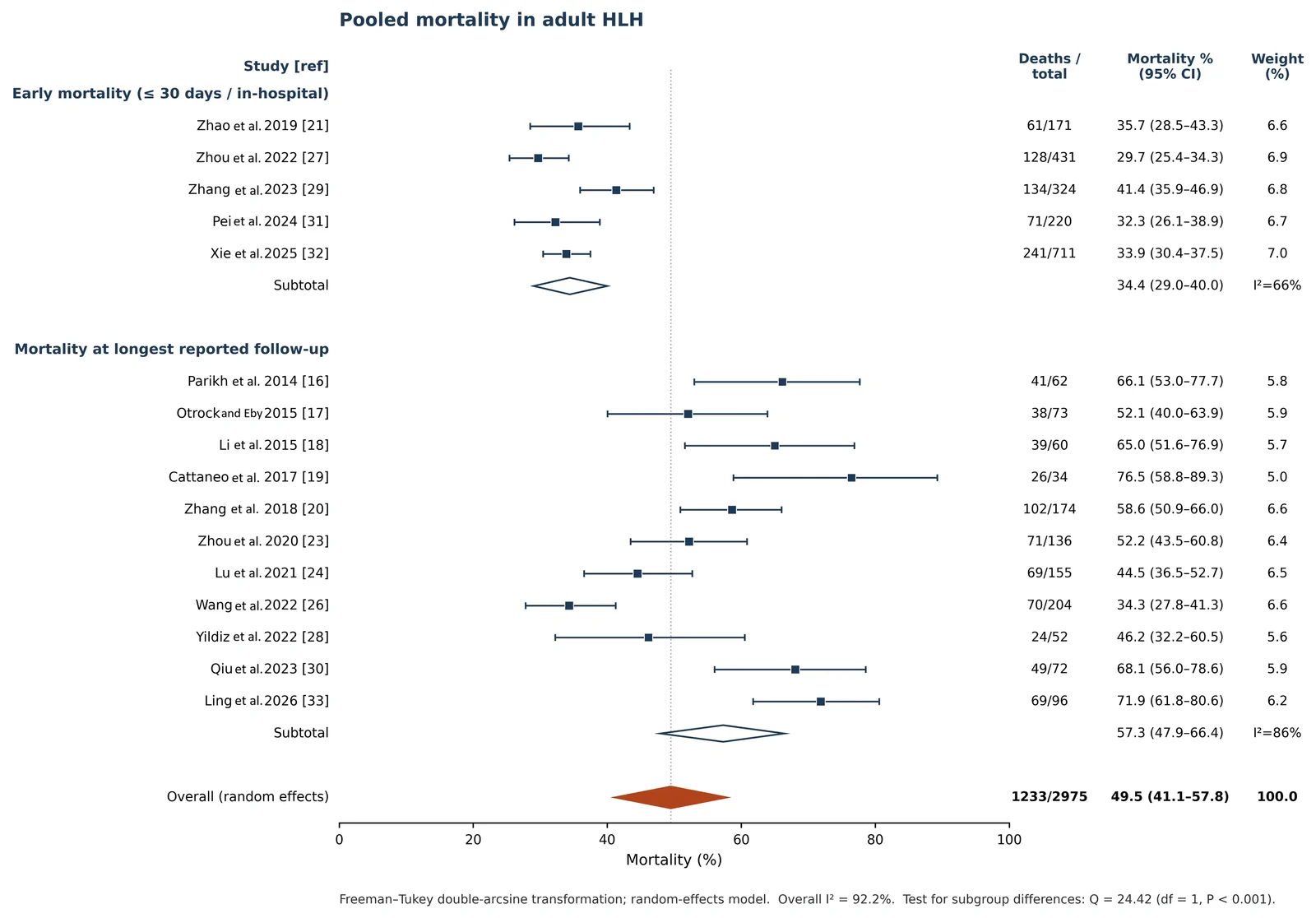
Pooled mortality in adult HLH, stratified by outcome window. HLH: hemophagocytic lymphohistiocytosis

A total of 16 cohorts reported extractable mortality counts (Figure 2). Pooled all-cause mortality was 49.5% (95% CI: 41.1-57.8), with substantial heterogeneity (I²=92.2%). Stratification by outcome window resolved a large part of this inconsistency and yielded a clinically informative gradient, as follows: pooled early mortality, defined as death within 30 days or during the index admission, was 34.4% (95% CI: 29.0-40.0; five cohorts; I² = 65.6%), whereas pooled mortality at the longest reported follow-up was 57.3% (95% CI: 47.9-66.4; 11 cohorts; I² = 86.4%). The difference between strata was significant (Q=24.42, df=1, p<0.001). Expressed differently, of every 100 adults given a diagnosis of HLH, approximately 34 are dead within a month, and approximately 57 are dead by the end of observation. This means that although the syndrome kills fastest in its opening weeks, a further quarter of the cohort is lost thereafter, most often because of progression of the underlying disease.

Malignancy-Associated HLH and Mortality

**Figure 3:**
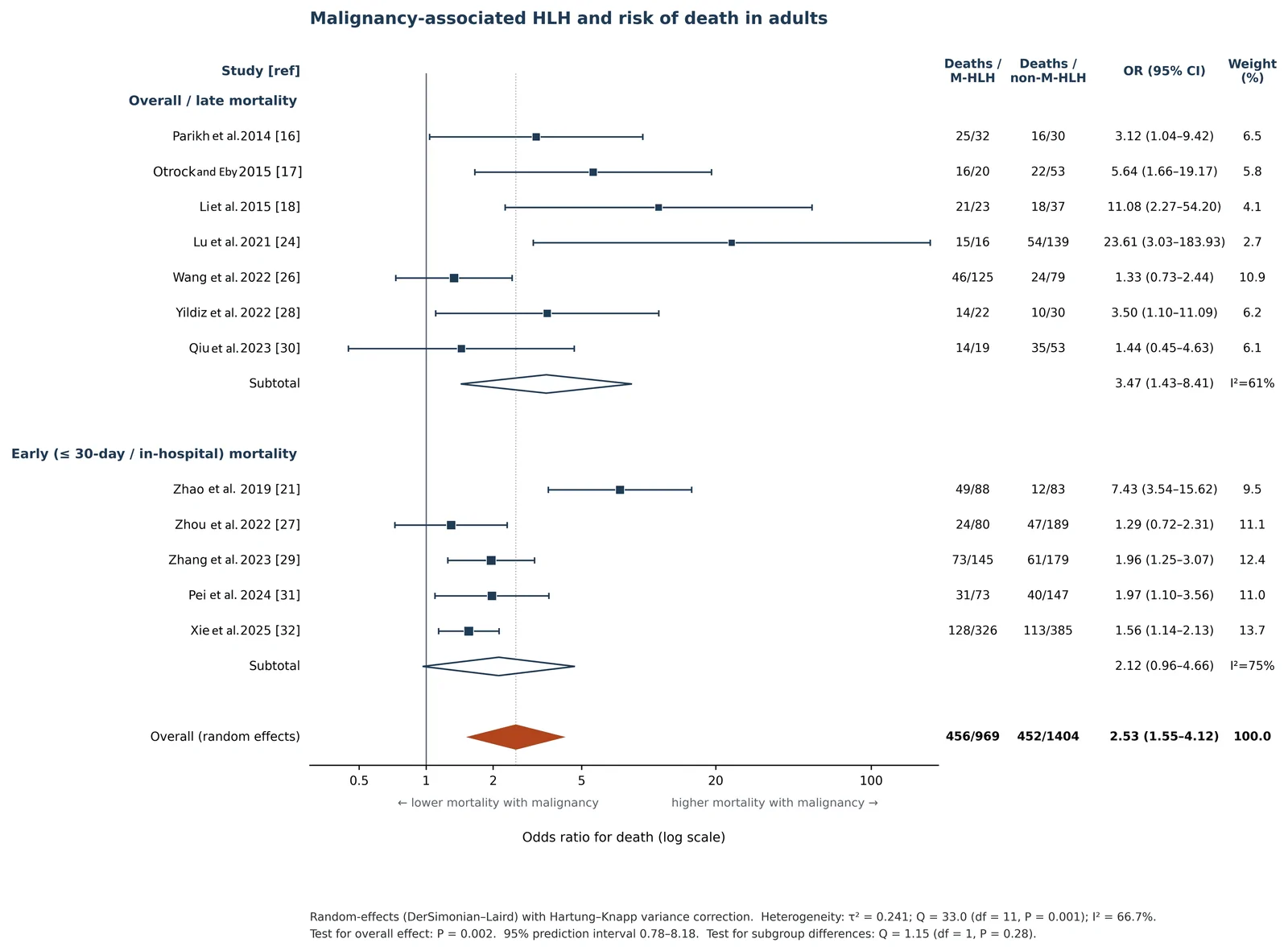
Forest plot of the association between a malignant trigger and death in adult HLH. HLH: hemophagocytic lymphohistiocytosis

A total of 12 cohorts, together enrolling 2,373 adults, permitted construction of a two-by-two table of deaths by trigger category (Figure 3, Table 4). In aggregate, 456 of 969 patients with malignancy-associated HLH died (47.1%), compared with 452 of 1,404 patients whose HLH was triggered by infection, autoimmunity, or an unidentified cause (32.2%). The pooled unadjusted odds ratio for death was 2.53 (95% CI: 1.55-4.12; p=0.002), with substantial between-study inconsistency (τ²=0.241; Q=33.00, df=11, p<0.001; I²=66.7%). Every one of the 12 point estimates lay above unity, ranging from 1.29 to 23.61; the direction of effect was therefore entirely uniform, and the heterogeneity resides in the magnitude rather than the sign of the association [24,27]. The 95% prediction interval, however, was wide (0.78-8.18), indicating that in a new setting the effect could plausibly be null.

**Table 4:** Deaths by underlying trigger and study-level odds ratios. HLH: hemophagocytic lymphohistiocytosis

Studies	Deaths/malignancy-associated HLH	Deaths/non-malignancy-associated HLH	Odds ratio (95% CI)	Weight (%)
Parikh et al. (2014) [16]	25/32 (78.1%)	16/30 (53.3%)	3.12 (1.04-9.42)	6.5
Otrock and Eby (2015) [17]	16/20 (80.0%)	22/53 (41.5%)	5.64 (1.66-19.17)	5.8
Li et al. (2015) [18]	21/23 (91.3%)	18/37 (48.6%)	11.08 (2.27-54.20)	4.1
Zhao et al. (2019) [21]	49/88 (55.7%)	12/83 (14.5%)	7.43 (3.54-15.62)	9.5
Lu et al. (2021) [24]	15/16 (93.8%)	54/139 (38.8%)	23.61 (3.03-183.93)	2.7
Wang et al. (2022) [26]	46/125 (36.8%)	24/79 (30.4%)	1.33 (0.73-2.44)	10.9
Zhou et al. (2022) [27]	24/80 (30.0%)	47/189 (24.9%)	1.29 (0.72-2.31)	11.1
Yildiz et al. (2022) [28]	14/22 (63.6%)	10/30 (33.3%)	3.50 (1.10-11.09)	6.2
Zhang et al. (2023) [29]	73/145 (50.3%)	61/179 (34.1%)	1.96 (1.25-3.07)	12.4
Qiu et al. (2023) [30]	14/19 (73.7%)	35/53 (66.0%)	1.44 (0.45-4.63)	6.1
Pei et al. (2024) [31]	31/73 (42.5%)	40/147 (27.2%)	1.97 (1.10-3.56)	11.0
Xie et al. (2025) [32]	128/326 (39.3%)	113/385 (29.4%)	1.56 (1.14-2.13)	13.7
Pooled (random effects)	456/969 (47.1%)	452/1404 (32.2%)	2.53 (1.55-4.12)	100.0

Pre-specified subgroup analyses did not identify a statistically significant modifier of this association, although the point estimates are instructive. The pooled odds ratio was 2.30 (95% CI: 1.22-4.33; I²=71.7%) in the nine East Asian cohorts and 3.87 (95% CI: 0.89-16.77; I²=0%) in the three non-Asian cohorts (Q=1.41, p=0.23). Stratifying by outcome window, the association was stronger for mortality at longest follow-up (OR: 3.47, 95% CI: 1.43-8.41; 7 cohorts) than for early mortality (OR: 2.12, 95% CI: 0.96-4.66; 5 cohorts), although the subgroup difference was not significant (Q=1.15, p=0.28), a pattern consistent with the biological expectation that a malignant trigger exerts its lethal effect progressively, through disease progression and treatment failure, rather than exclusively through the acute hyperinflammatory crisis. Smaller cohorts reported larger effects (OR: 3.59, 95% CI: 1.51-8.52 for n<150, vs. 2.17, 95% CI: 1.06-4.44 for n≥150; Q=1.39, p=0.24), a gradient that anticipates the finding on formal small-study-effect testing described below.

Adjusted Prognostic Factors

**Figure 4:**
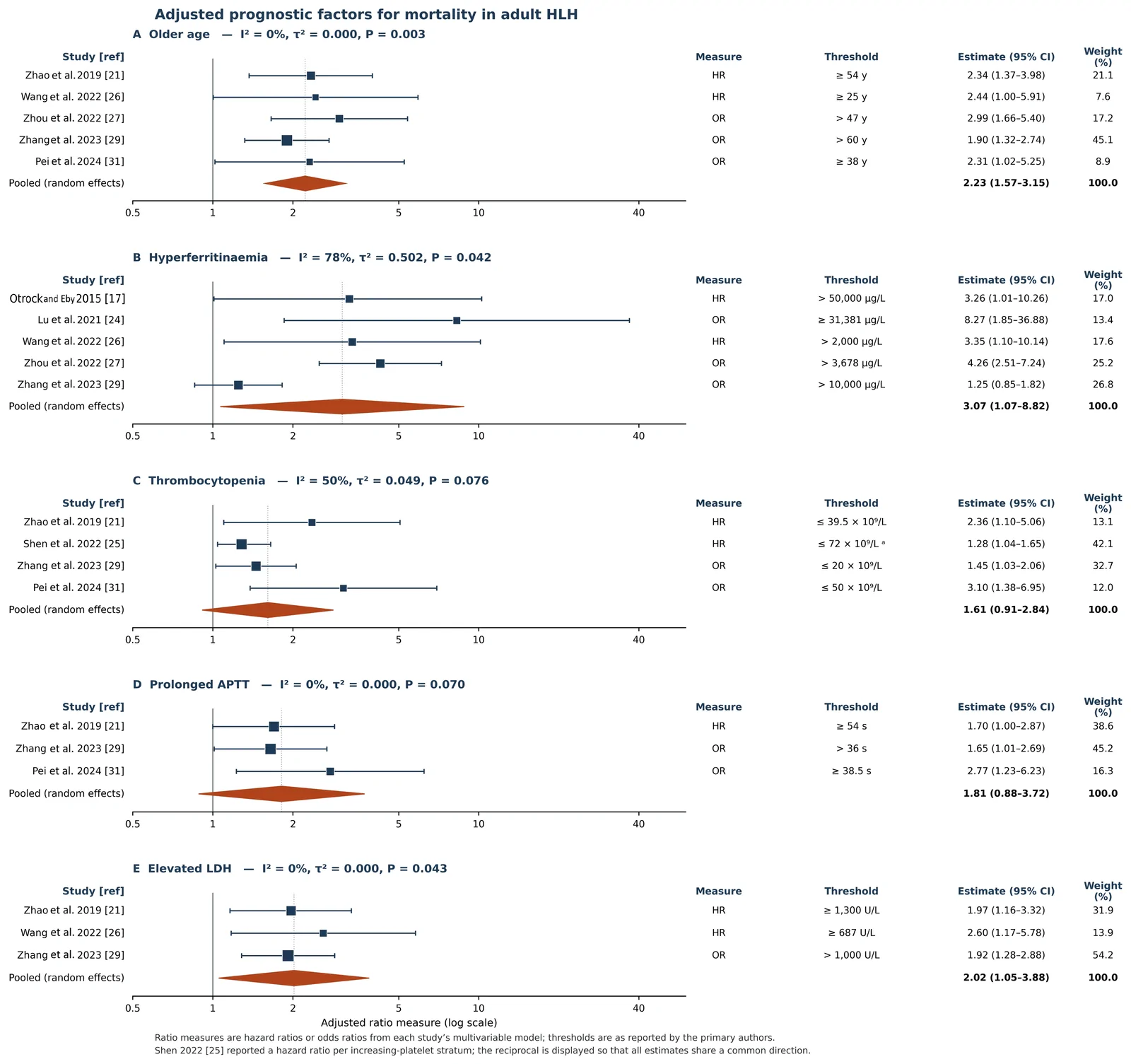
Forest plots of multivariable-adjusted prognostic factors for mortality in adult HLH. HLH: hemophagocytic lymphohistiocytosis; APTT: activated partial thromboplastin time; LDH: lactate dehydrogenase

Five factors met the pre-specified threshold of extractable multivariable-adjusted estimates in at least three independent cohorts (Figure 4). Older age was the most consistent predictor identified in this review as follows: pooled across five cohorts, the adjusted ratio measure for death was 2.23 (95% CI: 1.57-3.15; p=0.003), with no detectable inconsistency whatsoever (τ²=0.000; Q=1.76, df=4, p=0.78; I²=0%) and a prediction interval (1.50-3.31) that excluded the null. This homogeneity is striking given that the age thresholds applied in the contributing studies ranged from 25 to 60 years, and it suggests that the prognostic penalty of advancing age is robust to the precise dichotomy chosen.

Hyperferritinemia was significantly associated with death (five cohorts; pooled ratio 3.07, 95% CI: 1.07-8.82; p=0.042) but with substantial inconsistency (τ²=0.502; I²=78.4%) and a prediction interval spanning 0.24-39.69. Inspection of the contributing estimates makes the source of this heterogeneity plain as follows: the largest effect was reported by the study using the highest threshold (≥31,381 µg/L; ratio 8.27), whereas the only null estimate came from the cohort applying a threshold of >10,000 µg/L (ratio 1.25) [24,29]. Ferritin is thus not simply a dichotomous risk marker but a graded one whose prognostic value depends critically on where the line is drawn. Elevated lactate dehydrogenase was likewise a significant predictor (three cohorts; pooled multivariable-adjusted ratio: 2.02, 95% CI: 1.05-3.88; p=0.043) and, in contrast to ferritin, showed no heterogeneity at all (I²=0%), despite thresholds ranging from 687 to 1,300 U/L.

Thrombocytopenia (four cohorts; pooled ratio 1.61, 95% CI: 0.91-2.84; p=0.076; I²=50.2%) and prolonged activated partial thromboplastin time (three cohorts; 1.81, 95% CI: 0.88-3.72; p=0.070; I²=0%) were directionally consistent with harm in every contributing study but did not attain conventional significance under the Hartung-Knapp model. This loss of conventional significance is an artifact of the deliberately conservative Hartung-Knapp variance correction applied to analyses containing very few studies, rather than evidence of an absent association: under the classical DerSimonian-Laird model both associations were significant (thrombocytopenia 1.61, 95% CI: 1.17-2.21, p=0.003; prolonged activated partial thromboplastin time 1.81, 95% CI: 1.31-2.52, p<0.001), and the point estimates are identical. We report the Hartung-Knapp intervals as primary because they are the statistically defensible choice with three or four studies, but the honest conclusion for these two factors is “probably harmful, imprecisely estimated” rather than “not associated with death.” A summary of all six syntheses, with heterogeneity statistics and GRADE certainty ratings, is presented in Table 5.

**Table 5:** Summary of meta-analytic findings and GRADE certainty of evidence. ^a^The pooled estimate for the malignant trigger is an unadjusted odds ratio pooled from two-by-two tables, whereas the estimates for the other prognostic factors are pooled multivariable-adjusted ratio measures (combining hazard ratios and odds ratios). GRADE: Grading of Recommendations Assessment, Development, and Evaluation; APTT: activated partial thromboplastin time; LDH: lactate dehydrogenase

Prognostic factor	k	n	Pooled estimate (95% CI)ᵃ	p-Value	I² (%)	τ²	Certainty	Reason for downgrading
Malignant trigger	12	2,373	OR: 2.53 (1.55-4.12)	0.002	66.7	0.241	Low	Risk of bias (unadjusted estimates, residual confounding); publication bias (Egger p=0.014)
Older age	5	1,361	RR: 2.23 (1.57-3.15)	0.003	0.0	0.0	Moderate	Risk of bias (data-derived thresholds; heterogeneous adjustment sets)
Hyperferritinemia	5	1,258	RR: 3.07 (1.07-8.82)	0.042	78.4	0.502	Low	Risk of bias; inconsistency (I²=78%, threshold-dependent effect)
Thrombocytopenia	4	985	RR: 1.61 (0.91-2.84)	0.076	50.2	0.049	Low	Risk of bias; imprecision (CI includes the null)
Prolonged APTT	3	715	RR: 1.81 (0.88-3.72)	0.070	0.0	0.0	Low	Risk of bias; imprecision (k=3; CI includes the null)
Elevated LDH	3	699	RR: 2.02 (1.05-3.88)	0.043	0.0	0.0	Low	Risk of bias; imprecision (k=3; prediction interval crosses the null)
Pooled mortality (early)	5	1,857	34.4% (29.0-40.0)	-	65.6	-	Moderate	Inconsistency across settings
Pooled mortality (overall)	11	1,209	57.3% (47.9-66.4)	-	86.4	-	Low	Inconsistency; heterogeneous follow-up windows

Several additional factors were nominated as independent predictors by two cohorts each and, in accordance with the pre-specified rule, were not pooled as follows: hypoalbuminemia [16,25], prolonged prothrombin time [26,31], elevated creatinine [25,26], elevated aspartate aminotransferase, and elevated interleukin-10 [30-32]. Each was reported in the direction of harm, and hypoalbuminemia in particular retained independent significance in the Mayo Clinic cohort after adjustment for lactate dehydrogenase, ferritin, and malignant trigger [16]. These variables represent the most obvious candidates for the next generation of adult HLH prognostic research.

Sensitivity Analyses, Small-Study Effects, and Risk of Bias

Leave-one-out analysis of the malignancy synthesis confirmed that no single cohort drove the result as follows: the pooled odds ratio remained between 2.11 and 2.80 and remained statistically significant across all 12 iterations, with the lower confidence bound never falling below 1.36. Omission of the cohort with the greatest weight moved the pooled estimate to 2.80 (95% CI: 1.62-4.85), and omission of the most influential outlying estimate moved it to 2.11 (95% CI: 1.36-3.25) with a corresponding fall in I² from 66.7% to 50.1% [21,32]. Restricting the analysis to the seven cohorts of at least 150 patients yielded 2.17 (95% CI: 1.06-4.44), preserving significance.

**Figure 5:**
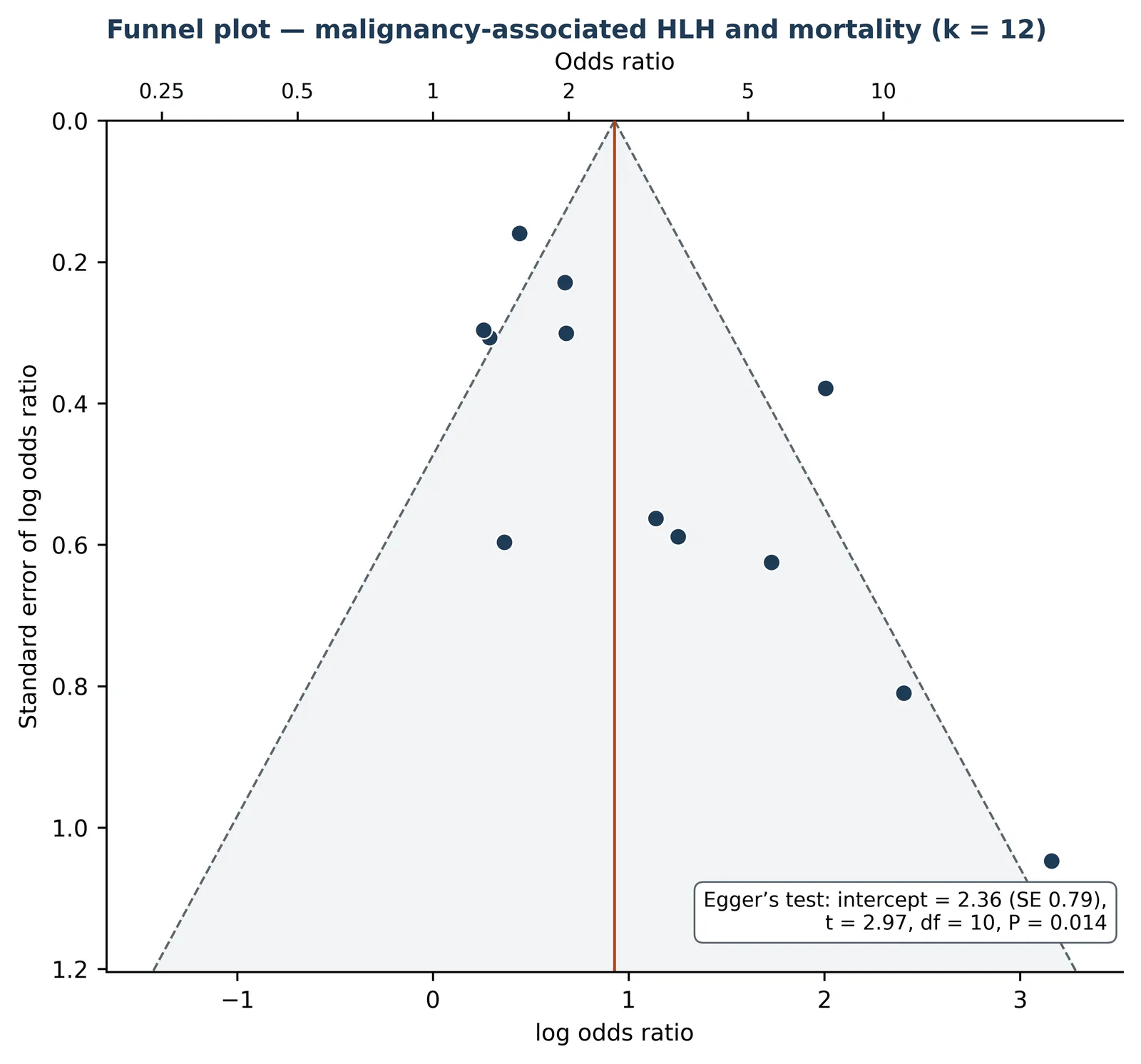
Funnel plot for the malignant-trigger analysis. HLH: hemophagocytic lymphohistiocytosis

The funnel plot for the malignancy analysis was visibly asymmetric, and Egger’s regression test confirmed this impression (intercept 2.36, standard error 0.80; t=2.97, df=10; p=0.014) (Figure 5). Smaller cohorts systematically reported larger odds ratios. Two explanations are plausible and are not mutually exclusive as follows: genuine publication or reporting bias, whereby small series demonstrating a striking effect of a malignant trigger were more likely to be written up and accepted; and true heterogeneity of effect, whereby small hematology-based series enroll a more aggressive lymphoma-dominated case-mix than large unselected hospital cohorts. Whichever mechanism predominates, the pooled odds ratio of 2.53 should be regarded as an upper bound rather than an unbiased estimate, and the more conservative figure of approximately 2.2 derived from the larger cohorts is the one we would carry into clinical reasoning.

Risk of bias is summarized in Table 6. Not one of the 18 cohorts achieved a low-risk rating in the study-confounding domain as follows: adjustment sets were selected by stepwise or data-driven procedures rather than being specified in advance on causal grounds, and no study adjusted for the intensity or timing of HLH-directed therapy, which is itself both a determinant of survival and a consequence of baseline severity. Prognostic factor measurement was rated high-risk in two cohorts because the candidate biomarker was measured after treatment had begun, at discharge, rather than at diagnosis - a design that is vulnerable to reverse causation, since patients who are dying of refractory disease cannot have a falling ferritin [22,23]. Attrition was a serious concern in only one cohort, in which follow-up data were unobtainable for 25 of 85 patients [18]. Statistical analysis and reporting were rated high risk across five cohorts due to a low events-per-variable ratio combined with extensive within-dataset cut-point optimization [18,19,22-24]. Study participation was rated low risk in the multicenter and consecutively enrolled cohorts and high risk in the smallest hematology ward series, in which four of the eight HLH-2004 criteria were unavailable [19,25,26,29,31,32].

**Table 6:** Risk-of-bias assessment using the Quality In Prognosis Studies (QUIPS) tool.

Studies	Study participation	Study attrition	Prognostic factor measurement	Outcome measurement	Study confounding	Statistical analysis and reporting
Parikh et al. (2014) [16]	Moderate	Low	Low	Low	Moderate	Moderate
Otrock and Eby (2015) [17]	Moderate	Low	Low	Low	Moderate	Moderate
Li et al. (2015) [18]	Moderate	High	Low	Moderate	High	High
Cattaneo et al. (2017) [19]	High	Moderate	Low	Low	High	High
Zhang et al. (2018) [20]	Moderate	Moderate	Low	Moderate	Moderate	Moderate
Zhao et al. (2019) [21]	Moderate	Low	Moderate	Low	Moderate	Moderate
Zhou et al. (2020) [22]	Moderate	Moderate	High	Low	High	High
Zhou et al. (2020) [23]	Moderate	Moderate	High	Low	High	High
Lu et al. (2021) [24]	Moderate	Moderate	Moderate	Low	Moderate	High
Shen et al. (2022) [25]	Low	Moderate	Moderate	Low	Moderate	Moderate
Wang et al. (2022) [26]	Low	Low	Moderate	Low	Moderate	Moderate
Zhou et al. (2022) [27]	Moderate	Low	Moderate	Low	Moderate	Moderate
Yildiz et al. (2022) [28]	Moderate	Low	Low	Low	Moderate	Moderate
Zhang et al. (2023) [29]	Low	Low	Moderate	Low	Moderate	Moderate
Qiu et al. (2023) [30]	Moderate	Moderate	Moderate	Moderate	Moderate	Moderate
Pei et al. (2024) [31]	Low	Low	Moderate	Low	Moderate	Moderate
Xie et al. (2025) [32]	Low	Low	Moderate	Low	Moderate	Moderate
Ling et al. (2026) [33]	Moderate	Moderate	Moderate	Moderate	Moderate	Moderate

Applying GRADE for prognostic factors, certainty was rated moderate for older age (downgraded once for risk of bias) and low for the malignant trigger (downgraded for risk of bias and for publication bias), hyperferritinemia (risk of bias, inconsistency), thrombocytopenia (risk of bias, imprecision), prolonged activated partial thromboplastin time (risk of bias, imprecision), and elevated lactate dehydrogenase (risk of bias, imprecision). Inconsistency was not counted against the malignancy analysis despite an I² of 66.7%, because all 12 point estimates lay on the same side of the null and the heterogeneity was one of magnitude alone. Full justifications are given in Table 5.

Discussion

Principal Findings

This systematic review and meta-analysis is, to our knowledge, the first to quantitatively pool prognostic factors for mortality in adult hemophagocytic lymphohistiocytosis. Drawing on 18 cohorts and 3,570 patients, it establishes three findings of immediate clinical relevance. First, adult HLH remains a lethal disease as follows: pooled mortality was 49.5%, with roughly one in three patients dead within 30 days of diagnosis and more than half dead by the end of observation. Second, the nature of the underlying trigger is the single most reproducible determinant of that outcome. A malignant trigger was associated with an approximate doubling of the odds of death (pooled odds ratio 2.53), and - crucially - every one of the 12 contributing cohorts pointed in the same direction, from Rochester to Chengdu to Nanjing [16,26,32]. Third, among variables measurable in any hospital laboratory on the day of diagnosis, older age, hyperferritinemia, and elevated lactate dehydrogenase carried independent prognostic information, whereas thrombocytopenia and prolonged activated partial thromboplastin time were directionally harmful but imprecisely estimated. What the analysis conspicuously failed to find was a single variable, or even a compact set of variables, precise and consistent enough to be trusted as a treatment-stratification instrument for an individual patient.

Reconciling a Discordant Mortality Literature

The pooled mortality figure deserves a moment’s attention because it reconciles a literature that has long appeared contradictory. Reported adult HLH case-fatality has been quoted as anything from 26.5% to 74.8%, and this range has been treated as an unexplained curiosity [28]. Our stratified analysis shows that a substantial part of it is simply an artifact of the observation window. Cohorts reporting 30-day or in-hospital mortality converged tightly on approximately 34%, whereas cohorts reporting mortality at longest follow-up converged on approximately 57%, and the difference between the strata was highly significant. The residual heterogeneity within each stratum then tracks case-mix as follows: hematology-ward series enriched for lymphoma, such as the Italian cohort in which 69% of patients had an underlying lymphoproliferative disorder, and 76% died [19], sit at the pessimistic extreme, whereas rheumatology-facing cohorts in which macrophage activation syndrome predominates sit at the optimistic one [31]. The practical message is that a clinician quoting a prognosis to a family should specify the horizon as follows: the first month is the period of maximum hazard, but survival of that month is not equivalent to cure, since a further quarter of the cohort is lost subsequently, most often to progression of the underlying malignancy rather than to hyperinflammation itself.

The Malignant Trigger Is the Dominant Determinant of Outcome

The dominance of the malignant trigger is biologically coherent and is corroborated by evidence beyond the studies we pooled. In the Mayo Clinic series, median overall survival was 1.4 months in tumor-associated HLH vs. 22.8 months in non-tumor-associated disease [16]; in the Washington University cohort, malignancy was the only independent predictor of poor survival on multivariable analysis, with a hazard ratio of 12.22 [17]. Neither of those cohorts is East Asian, and neither is large, yet the effect is unmistakable. The mechanistic explanation is that in lymphoma-associated HLH the neoplastic clone is itself the engine of the cytokine storm - neoplastic T cells secrete interferon-γ and other mediators directly, while in B-cell lymphoma the surrounding activated T-cell infiltrate does so - with the consequence that immunosuppression alone cannot extinguish the syndrome unless the tumor is simultaneously controlled. This is precisely why the outcome of lymphoma-associated HLH tracks so closely with response to anti-lymphoma therapy [20], and why the histological subtype matters so much - multiple included cohorts found that natural killer/T-cell lymphoma carried the worst prognosis of all, and that B-cell disease, being far more chemosensitive in the rituximab era, was associated with materially better survival [19,20]. Our subgroup analysis, showing a numerically stronger malignancy effect on overall than on early mortality, is entirely consistent with this model as follows: the malignant trigger kills progressively, through treatment failure, rather than solely through the acute inflammatory crisis.

Corroboration From Studies Not Eligible for Inclusion

Placing these findings in the context of studies not eligible for inclusion strengthens rather than weakens them. Arca et al., in a French cohort of 162 adults with reactive hemophagocytic syndrome, found that a malignant trigger and the absence of early etoposide were the principal determinants of early death - a finding directly parallel to ours, and one that raises the therapeutic corollary that the prognostic penalty of malignancy may be partially modifiable [34]. The multicenter North American series of Schram et al. likewise identified underlying malignancy as the dominant driver of mortality in adults and reported that platelet count and alanine aminotransferase were independent determinants of overall survival, echoing the platelet signal that we pooled [35]. Birndt et al., reporting the German nationwide registry of 137 adults, found the same trigger-dependent gradient of outcome [36]. Zoref-Lorenz et al. went further and constructed an optimized diagnostic and prognostic index specifically for malignancy-associated HLH, demonstrating that within that sub-population soluble CD25 and ferritin carry independent mortality information - a study we deliberately excluded because it is restricted to a single trigger, but which usefully indicates the direction that adult HLH prognostication ought now to take [37]. Finally, the large United States administrative-database analysis of Abdelhay et al., spanning 2006-2019, documented an in-hospital mortality that has barely improved across more than a decade [38], and the long-term follow-up of the international HLH-2004 study demonstrated that even in the population for which the protocol was designed, etoposide-based therapy achieves only partial success [39]. Taken together, these external data support the central conclusion of our synthesis as follows: the trigger, rather than the intensity of the hyperinflammatory phenotype, is what primarily determines whether an adult with HLH survives.

Data-Derived Thresholds and Their Consequences

The behavior of ferritin in our analysis merits particular scrutiny, because it exposes a methodological pathology that runs through this entire literature. Ferritin was significantly associated with death when pooled (ratio: 3.07), yet the heterogeneity was substantial (I²=78.4%), and the prediction interval spanned two orders of magnitude. The explanation is not statistical noise but the thresholds themselves. The strongest effect came from the study using a cut-point of 31,381 µg/L [24]; the null effect came from the study using 10,000 µg/L [29]; and the studies using 2,000, 3,678 and 50,000 µg/L fell in between with no coherent ordering [17,26,27]. These cut-points were not chosen on biological grounds. They were derived, in every case, by optimizing a receiver operating characteristic curve or a maximally selected rank statistic within the very dataset on which they were later tested. A threshold obtained this way is guaranteed to perform well in its parent cohort and is under no obligation to perform anywhere else, and the near-certainty that it will not transfer is the reason why a decade of adult HLH prognostic modeling has produced a dozen mutually incompatible nomograms and no clinically adopted score. The same criticism applies with equal force to the age thresholds, which ranged from 25 to 60 years across the five pooled cohorts, and to the platelet, lactate dehydrogenase, and coagulation cut-points. That older age nonetheless emerged with an I² of exactly zero, despite this threshold chaos, is a genuinely reassuring result as follows: it indicates that the prognostic gradient of age is strong enough to survive arbitrary dichotomization, and it suggests that age should be modeled as a continuous variable in any future index rather than being forced into a category.

Confounding by Indication and the Timing of Biomarker Measurement

Two further observations from the risk-of-bias assessment bear directly on how these pooled estimates should be read. The first concerns confounding. Not a single one of the 18 cohorts achieved a low-risk rating in this domain, and the reason is structural - no study adjusted for HLH-directed therapy, which is both a determinant of survival and a consequence of baseline severity. Patients who are moribund at presentation frequently receive supportive care alone, and the resulting association between “no etoposide” and death is at least partly confounded by indication. The very large Nanjing cohort makes this vivid - supportive care alone was associated with a 48.2% 30-day survival against 78.4% for HLH-specific therapy - but no causal inference is possible from that contrast, and none of the prognostic estimates we pooled is adjusted for it [32]. The second concerns the timing of biomarker measurement. Two included studies evaluated ferritin at discharge rather than at diagnosis, which inverts the direction of inference as follows: a patient dying of refractory HLH cannot have a falling ferritin, so a high post-treatment ferritin is as much a marker of impending death as a predictor of it [22,23]. We excluded those estimates from the ferritin pool for precisely this reason (as now explicitly marked in Table 3), and we would urge future investigators to distinguish sharply between baseline prognostic markers and dynamic response markers, which address entirely different clinical questions.

Small-Study Effects

The finding of small-study effects in the malignancy analysis (Egger p=0.014) must be reported honestly and interpreted carefully. Smaller cohorts systematically reported larger odds ratios, and the pooled estimate from the seven cohorts of at least 150 patients was 2.17 rather than 2.53. Publication bias is one plausible explanation, and in a literature composed almost entirely of single-center retrospective series it would be surprising if it were absent. But a second, equally plausible explanation is that small series are disproportionately drawn from hematology wards, where the case mix is dominated by aggressive lymphoma, whereas the largest cohorts are unselected, hospital-wide series in which milder infection- and autoimmunity-associated disease dilutes the malignancy signal. The two mechanisms cannot be distinguished from the available data, and we therefore regard the pooled odds ratio of 2.53 as an upper bound and the figure of approximately 2.2 from the larger cohorts as the more defensible estimate for clinical reasoning. Either way, the direction and the clinical implication are unaltered as follows: an adult with HLH and an underlying malignancy is at substantially higher risk of death, and the search for the tumor should be pursued with the same urgency as the treatment of the hyperinflammation.

Subgroups, Triggers, and Biomarkers That Could Not Be Pooled

Several clinically important questions could not be answered with the available aggregate data, and each one defines a priority for future work. First, we did not separate primary (familial) from secondary (acquired) disease; this reflects the biology of the adult population rather than an analytic omission, because every included cohort was composed overwhelmingly or exclusively of secondary HLH and no cohort reported a genetically confirmed primary subgroup that could be analyzed apart. Second, malignancy-associated HLH was of necessity treated as a single category, because only a minority of cohorts reported deaths separately for lymphoma, leukemia, and solid tumors; where subtype was reported, natural killer/T-cell lymphoma carried the worst prognosis and chemosensitive B-cell disease the best, a gradient we describe qualitatively but could not quantify [19,20]. Third, infection-associated disease could not be resolved by pathogen as follows: only single cohorts identified specific organisms as independent predictors, Epstein-Barr virus and fungal infection in the Nanjing cohort, for example, and these did not meet the three-cohort threshold for pooling, so the recognized prognostic difference between EBV-associated and other infectious HLH could not be captured [31]. Fourth, a separate pooled estimate could not be generated for autoimmune/macrophage activation syndrome-predominant disease. The cohorts in which this condition predominated were at the more favorable end of the mortality range, consistent with its generally better prognosis. However, the primary studies did not report trigger-specific effect estimates that could be combined [31]. Fifth, the impact of treatment timing, in particular early etoposide, on mortality could not be quantified, because no included cohort reported it as an adjusted, poolable effect; its likely importance, and the confounding by indication that obscures it, are discussed above and are supported by the external French cohort [34]. Sixth, newer biomarkers such as soluble CD25/soluble interleukin-2 receptor, natural killer cell activity, CXCL9, and cytokine panels were either unavailable in most of the contributing (predominantly Chinese) centers or reported by single cohorts only (interleukin-6 [24], interleukin-10 [30]), so none could be pooled, even though external data indicate that soluble CD25 carries independent prognostic weight in malignancy-associated disease [37]. Seventh, markers of organ dysfunction, neurological involvement, hepatic and renal failure, and intensive-care admission were reported heterogeneously (as altered mental status, bilirubin, creatinine, and edema within individual models) and could not be harmonized into a single pooled analysis (Table 3). Eighth, the included studies did not report head-to-head prognostic comparisons of the HLH-2004 criteria against the HScore, so the relative prognostic performance of the two instruments remains an open question. Finally, we could not examine the interaction between the underlying trigger and individual biomarkers - for example, whether hyperferritinemia carries the same prognostic weight in malignancy as in infection-associated disease - because the primary studies did not report trigger-stratified biomarker effects. Each of these gaps is a direct consequence of the fragmented, single-center, aggregate-data literature, and each argues for the prospective, individual-participant-data registry we advocate below.

Clinical Implications

What, then, should a clinician take from this synthesis? Not a score, and not a threshold. Rather, a hierarchy of prognostic weight. The trigger comes first - identifying or excluding an underlying malignancy is the single most informative prognostic act, and it is also the most actionable, since lymphoma-associated HLH demands rapid anti-lymphoma therapy rather than immunosuppression alone. Age comes second, and should be treated as a continuous gradient rather than a cliff. The laboratory markers - ferritin, lactate dehydrogenase, platelet count, and the coagulation indices - come third, and are best understood as indices of the severity of the hyperinflammatory state rather than as independent, transferable predictors with defensible cut-points. And what the field needs next is not another single-center nomogram. It is a prospective, multinational adult HLH registry with pre-specified continuous predictors, a pre-registered analysis plan, mandatory reporting of HLH-directed therapy as a time-varying covariate, and external validation in a cohort that had no part in the model’s derivation. Until such a study exists, the estimates presented here, pooled, transparent, and explicitly graded as low to moderate certainty, represent the best available quantitative summary of a literature that has, so far, generated more models than knowledge.

Limitations

This review has important limitations, most of which are inherited from the primary literature. The review was not prospectively registered with PROSPERO. Although the protocol, the eligibility criteria, and the rule requiring at least three cohorts before pooling were all fixed before data extraction began, the absence of a public registration means that readers cannot independently verify that no post-hoc changes were made, and the possibility of selective reporting at the review level cannot be formally excluded. All 18 included studies were observational, and 17 were retrospective, so residual confounding is unavoidable, and no causal interpretation of any association is warranted. Fifteen of the 18 cohorts were Chinese, which restricts generalisability as follows: the trigger distribution in East Asia is malignancy-dominant, whereas infection predominates in several Western series, and the pooled estimates may therefore over-represent lymphoma-associated disease. Soluble CD25 and natural killer cell activity assays were unavailable at most Chinese centers, so diagnosis in those cohorts relied on a reduced set of criteria, introducing an ascertainment difference we could not quantify. We combined hazard ratios and odds ratios on the log scale as generic ratio measures; because mortality in this population is far from rare, these are not numerically equivalent, and this approximation will have introduced some bias, most plausibly away from the null for the odds-ratio-based studies, since with a common outcome the odds ratio is more extreme than the corresponding risk ratio. Cut-points for every continuous prognostic factor were data-derived and mutually incompatible, so the pooled estimates for ferritin, platelets, lactate dehydrogenase, and the coagulation indices should be interpreted as answering the question, “Does an abnormal value predict death?” rather than, “Is this specific threshold clinically useful?”. For the same reason, due to the reliance on aggregate, non-stratified data, we were unable to stratify malignancy-associated disease by tumor type, to resolve infection-associated disease by pathogen, to analyze macrophage activation syndrome separately, to compare the prognostic performance of the HLH-2004 criteria against the HScore, or to test for interaction between the trigger and individual biomarkers. Adjustment sets differed between studies, and none was pre-specified; no study adjusted for HLH-directed therapy. Three of the six meta-analyses included only three or four cohorts, for which between-study variance is poorly estimated; we mitigated this using the Hartung-Knapp correction, but the resulting intervals are necessarily wide. Publication bias could be formally assessed for only one analysis, and it was detected.

## Conclusions

Approximately half of all adults diagnosed with hemophagocytic lymphohistiocytosis die, and approximately one-third die within the first 30 days. An underlying malignancy is the most reproducible prognostic factor identified in this synthesis, approximately doubling the odds of death across every cohort examined, and older age, hyperferritinemia, and elevated lactate dehydrogenase provide additional independent prognostic information. Thrombocytopenia and coagulopathy are directionally harmful but too imprecise to rely on. The certainty of this evidence is low to moderate, limited chiefly by residual confounding, by the absence of any adjustment for HLH-directed therapy, and by the pervasive use of within-dataset threshold optimization that renders published cut-points non-transferable. These findings support an immediate and exhaustive search for an occult malignancy in every adult presenting with HLH, and they argue that the next advance in adult HLH prognostication will come not from another single-center nomogram but from a prospective, multinational registry with pre-specified continuous predictors and genuine external validation.
